# Reducing bias through directed acyclic graphs

**DOI:** 10.1186/1471-2288-8-70

**Published:** 2008-10-30

**Authors:** Ian Shrier, Robert W Platt

**Affiliations:** 1 Centre for Clinical Epidemiology and Community Studies, SMBD-Jewish General Hospital, McGill University, Montreal, Canada; 2 Department of Epidemiology and Biostatistics, McGill University, Montreal, Canada

## Abstract

**Background:**

The objective of most biomedical research is to determine an unbiased estimate of effect for an exposure on an outcome, i.e. to make causal inferences about the exposure. Recent developments in epidemiology have shown that traditional methods of identifying confounding and adjusting for confounding may be inadequate.

**Discussion:**

The traditional methods of adjusting for "potential confounders" may introduce conditional associations and bias rather than minimize it. Although previous published articles have discussed the role of the causal directed acyclic graph approach (DAGs) with respect to confounding, many clinical problems require complicated DAGs and therefore investigators may continue to use traditional practices because they do not have the tools necessary to properly use the DAG approach. The purpose of this manuscript is to demonstrate a simple 6-step approach to the use of DAGs, and also to explain why the method works from a conceptual point of view.

**Summary:**

Using the simple 6-step DAG approach to confounding and selection bias discussed is likely to reduce the degree of bias for the effect estimate in the chosen statistical model.

## Background

The objective of most biomedical research, whether experimental or observational, is to predict what will happen to an outcome if the treatment is applied to a group of individuals or if a harmful exposure is removed. In other words, the clinician/policy maker is interested in making causal inferences from the results of a study. The purpose of this manuscript is to demonstrate a simple 6-step algorithm for determining whether a proposed set of covariates would reduce possible sources of bias when assessing the total causal effect of a treatment on an outcome.

There are many nuances to the definition of cause. For the purposes of this manuscript, we define it in counterfactual terms: "Had the exposure differed, the outcome would have differed", where exposure or outcome may be dichotomous (e.g. presence/absence of exposure; occurrence/disappearance of disease) or continuous (e.g. a different value of blood pressure whether blood pressure is exposure or outcome). Further refinements into sufficient, complementary and necessary causes [1] are important but do not alter the essence of the definition. Although the above causal definition is deterministic at the individual level, in almost all practical settings the outcome under the counterfactual condition is unknown. Therefore, researchers are limited to causal inference at the population level (e.g. comparing average risks) [2]. A straightforward explanation of the use of counterfactuals to define cause can be found in [2].

**Figure 1:**
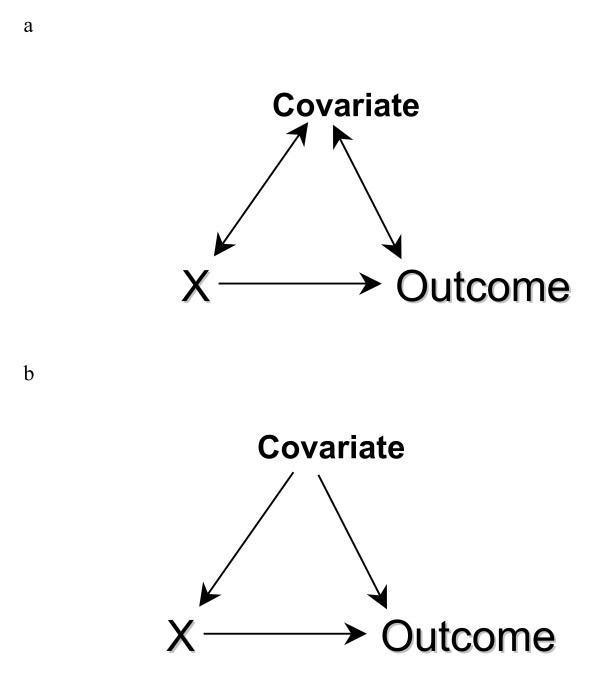
**The bi-directional arrows in A show the traditional representation of a confounder as being associated with the exposure (X) and outcome. **Because confounders must cause (or be a marker for a cause) of both exposure and outcome (see text for rationale based on basic principles), directed acyclic graphs use only unidirectional arrows to show the direction of causation (B).

There are many features of a study that can lead to inappropriate causal inference. For the purposes of this discussion, we assume "ideal" processes for the study (i.e. large studies that minimize the risk of a chance unequal distribution of subjects with different prognoses, no information or selection or detection bias, complete follow-up and adherence, no measurement bias, etc). Under "ideal" conditions, inappropriate causal inferences (i.e. biases) are more likely to occur in observational studies compared to randomized trials because some subjects may be exposed to a treatment for a condition specifically because of personal factors that are related to prognosis (figure 1a). Under the conditions described above, most epidemiologists would consider this confounding bias. We recognize that there are several definitions of confounding bias and Greenland and Morgenstern provide an excellent overview of the nuances among the different definitions [3].

The traditional approach to confounding is to 'adjust for it' by including certain covariates in a multiple regression model (or by stratification). One common practice is to consider a covariate to be a confounder (and "adjust" for it) if it is associated with the exposure, associated with outcome, and changes the effect estimate when included in the model. According to standard textbooks, additional criteria also need to be applied and the covariate should not be affected by exposure and needs to be an independent cause of the outcome [4]. However, recent advances in epidemiology have proven that even these additional criteria are insufficient. In fact, the methods described above may introduce conditional associations (sometimes called selection bias [5,6], collider bias [6] and confounding bias [6,7]; this terminology may be confusing and we prefer the terminology suggested by the structural approach to bias as described later) and create bias where none existed, which is in direct contrast to the objective of eliminating an existing bias [2,8,9]. Some published examples include the effectiveness of HIV treatment [10], and why birth weight should not be included as a covariate when examining the causal effects of exposure during pregnancy on perinatal outcomes [11].

One method to help understand whether bias is potentially reduced or increased when conditioning on covariates is the graphical representation of causal effects between variables. In the causal directed acyclic graph (DAG) approach, an arrow connecting two variables indicates causation; variables with no direct causal association are left unconnected. Therefore the bi-directional arrows in figure 1a are replaced with unidirectional arrows (figure 1b). There are of course situations where each variable may cause the other – the functional disability created by chronic pain may cause depression, and depression may cause chronic pain through diminished pain thresholds. These more complex situations are simplified by understanding that time is a component in the above relationship. Therefore, there is a variable for depression at time 1, chronic pain at time 1, depression at time 2, and chronic pain at time 2; the same construct measured at different times represents distinct variables and must be treated as such.

Although other articles have previously described the DAG approach to confounding [9,12,13], the articles demonstrate relatively simple DAGs. However, many clinical problems require complicated DAGs and little has been published on how to assess whether a particular subset of covariates potentially reduces or increases bias in this context [6,9]. Therefore, although many investigators now understand the problem, they continue to use traditional practices because they do not have the tools necessary to choose the statistical model that is most likely to yield an unbiased effect estimate. The objective of this manuscript is to demonstrate a simple 6-step approach developed by Pearl [14] that helps determine when the traditional statistical approaches of regression/stratification on specific covariates is likely to reduce or increase bias, and to provide our explanation as to why the method works from a conceptual point of view. Although this manuscript is limited to the conceptual discussion necessary for clinical researchers to use DAGs, there are many different facets and a complete theoretical development of these materials has been published elsewhere and has been summarised in one source [15]. Readers are also encouraged to learn more about counterfactual random variables, an important complement to the theory of DAGs [3,16].

### The Pragmatic Solution: a Six-Step Process Towards Unbiased Estimates [14]

**Figure 2:**
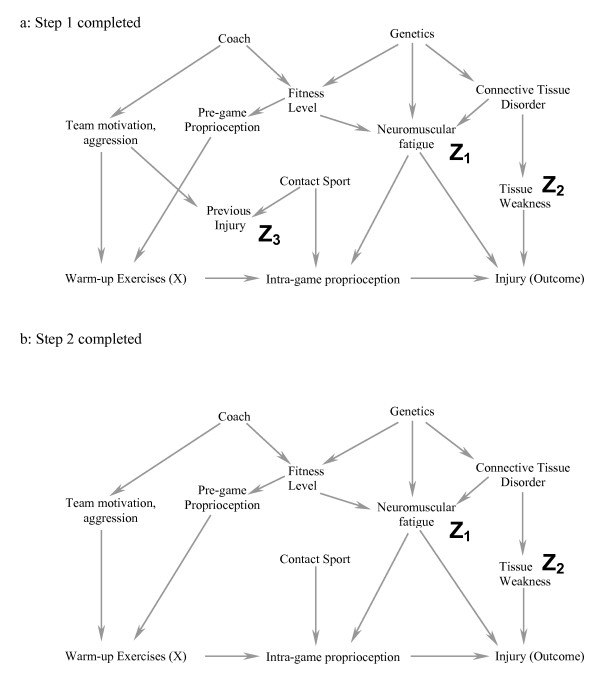
**a-b. Diagrammatic equivalent of the 6-step process to determine if one obtains an unbiased estimate of the exposure of interest (X) on the Outcome by including a particular subset of covariates (see text for details of the specific steps).** In this example, we are interested in minimizing the bias when estimating the causal effect of warming up on the risk of injury. In figure 2a, a possible causal diagram of variables that are associated with warming up (X) and injury (outcome) are shown. The main mediating variable is believed to be proprioception (balance and muscle-contraction coordination) during the game. Starting at the top of the figure, the coach affects the team motivation (including aggressiveness), which affects both the probability of previous injury and the player's compliance with warm-up exercises. A player's genetics affects their fitness level (along with the coach's fitness program) and whether there are any inherent connective tissue disorders (which leads to tissue weakness and injury). Both connective tissue disorders and fitness level affect neuromuscular fatigue, which independently affects proprioception during the game and the probability of injury. Finally, if the sport is a contact sport, the probability of previous injury is greater, as is the probability of minor bruises during the game that would affect proprioception. Although other causal models are also possible, we will use this one for illustrative purposes at this time. For this example, we have decided to include neuromuscular fatigue (Z_1_) and tissue weakness (Z_2_) in the statistical model. Step #1 is to ensure that these covariates are not descendants of (i.e. directly or indirectly caused by) warm-up exercises. Step 2 is illustrated in 2b. The open circle (previous injury, Z_3_) represents the only non-ancestor (an ancestor is direct or indirect cause of another variable) of warm up exercises (X), neuromuscular fatigue (Z_1_), tissue weakness (Z_2_) and injury (Outcome). It is therefore deleted from the causal diagram in figure 2b.

By applying the following simple 6-step process correctly, we will show how including only 2 covariates in a complicated causal diagram (figure 2a) is likely to reduce bias. As each step is described, we also explain its conceptual role in the process. Formal proofs of the underlying theorems have been summarised in one source [15]. In the subsequent section of the manuscript, we will add an additional covariate from the diagram into the model and show how including this additional variable is likely to increase bias rather than reduce it. It is important to note that this algorithm demonstrates whether bias would be minimized in a specific situation, but does not indicate all the situations in which bias is minimized. For example, if a confounder causes a second variable with a high probability (i.e. the second variable is a strong marker for the confounder), including the marker for the confounder should reduce bias [6]. However, in this situation, the algorithm we describe would still suggest that there is bias in the effect estimate. Therefore, the algorithm is used to "rule-in" appropriate sets of covariates and it is beyond the scope of this article to discuss the special cases where bias might be reduced even when the algorithm fails. Therefore, if the algorithmic conditions are not met, readers are encouraged to either choose another set of covariates, or seek further help in order to determine if their particular model is one of the cases where bias might still be reduced.

Figure 2a is one possible causal diagram for the relationship between warming up prior to exercise and the outcome injury (we will show another possible causal diagram later in the manuscript). The question we want to answer is whether including a measure of neuromuscular fatigue (Z_1_) and tissue weakness (Z_2_) (in the design or analysis stage) would minimize bias in the estimate of the effect of warming up on injury if this is the true causal diagram. We will later discuss how to approach the more general problem when multiple causal diagrams are possible. As with any analytic approach to bias in an observational study (including the one below), we must make some assumptions regarding how variables are causally related to each other; we seek to determine whether our analytic approach would succeed under these assumptions. The algorithm we describe below only works if the DAGs are drawn so that they include all variables that cause two or more other variables shown in the DAG [17]. In other words, no common causes can be omitted from the DAG. Finally, the DAG approach does not reduce or eliminate other sources of bias (e.g. measurement bias). Finally, at the end of the manuscript, we have provided a glossary of terms used so that readers unfamiliar with DAG terminology have an easy reference immediately available (genealogical terms are often used to describe relationships between variables).

### Step 1 (figure 2a): The covariates chosen to reduce bias [fatigue (Z_1_) and tissue weakness (Z_2_) in this case] should not be descendants of X (i.e. they should not be caused by warming up)

#### This does not occur in this situation and one can proceed to Step 2

Step 1 ensures that the covariates chosen are possible confounders in the traditional sense of the word; if the covariates are descendants of X, then the statistical model adjusting for these variables may yield a biased estimate for the total causal effect of X on the outcome and a different set of covariates needs to be chosen. The step is required because confounding bias (as defined by the structural approach) can only occur if a covariate causes the exposure or is a marker for a cause of exposure (note that other biases are still possible and discussed in Step 4). Although more formal proofs exist [12], this can be deduced from the following standard criteria for a potential confounder: the covariate must be associated with the exposure and with the outcome, but cannot be affected (i.e. caused) by exposure [4,18] (for completeness, these standard criteria are in fact insufficient to define confounding [9], and more complicated scenarios such as time-dependent confounding [19] are not covered by the standard definitions). Because it is inappropriate to include a variable that lies along a causal pathway between the exposure of interest and the outcome, it is also inappropriate to include a marker for a variable that lies along a causal pathway. For example, if the marker is 100% correlated with the causal pathway variable, there is no mathematical difference in a statistical model between the marker and the causal pathway variable. Thus, if a covariate is associated with an exposure, and the exposure cannot cause or be a marker for a cause of the covariate, then the covariate must cause (or be a marker for a cause of) the exposure. By similar reasoning, one can deduce that confounding only occurs if the covariate also causes, or is a marker for a cause of the outcome. Returning to the DAG, if the covariate is a descendant of X, it means the exposure is a cause of the covariate.

### Step 2 (figure 2b): Delete all variables that satisfy all of the following: 1) non-ancestors (an ancestor is a variable that causes another variable either directly or indirectly) of X, 2) non-ancestors of the Outcome and 3) non-ancestors of the covariates that one is including to reduce bias (Z_1 _and Z_2 _in this example)

#### In figure 2a, the only covariate that fulfills this criterion is previous injury (Z_3_) and this is deleted in figure 2b. Note that the exposure, outcome and covariates should not be deleted

Step 2 is essential because after completing the step, all variables left are either conditioned on, or have one of their descendants conditioned on. The importance of this result will become clear in Step 4.

### Step 3 (figure 3a): Delete all lines emanating from X

**Figure 3:**
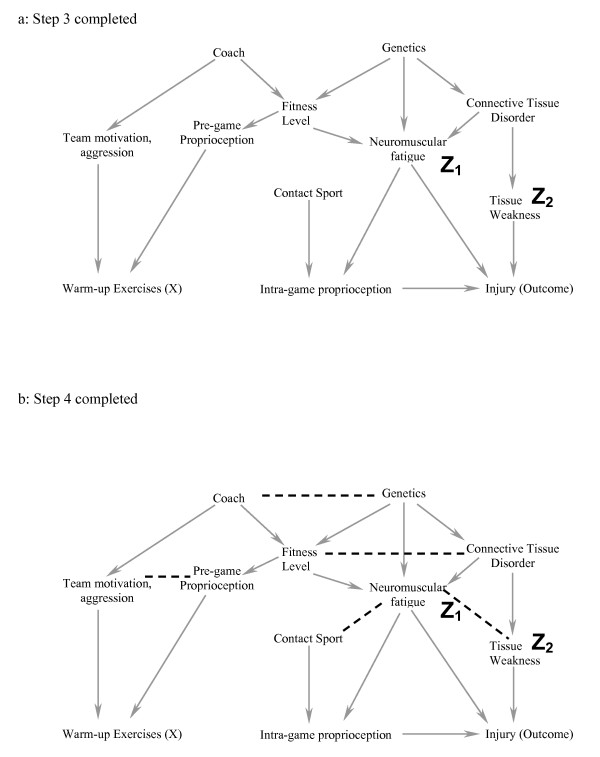
**a-b. In Step 3 (3a), all arrows emanating from X are deleted.** In Step 4 (3b), one joins all parents of a common child. We have used dashed lines here for clarity.

#### In this setting, warming-up causes a change in proprioception, and therefore we delete this arrow

In Step 3, deleting all lines emanating from X effectively simplifies the DAG because we have already said that X should not be a cause of the covariates in the model. We leave the variables in and eliminate the line because these variables may be responsible for bias through an indirect pathway. This will become clearer in Step 4, and the example where we include a third covariate, which results in the introduction of bias.

### Step 4 (figure 3b): Connect any two parents (direct causes of a variable) sharing a common child (this step appears simple but it requires practice not to miss any)

#### For example, team motivation and poor proprioception can both cause an individual to warm-up more than someone without these factors – these two variables are joined because they share a common effect

Step 4 is essential for the following reason. If two covariates both cause a third covariate, then adjustment for the third covariate (or an effect of the third covariate) creates a conditional association between the first two covariates (i.e. if one conditions on the child or descendant of the child, there is a conditional association between the parents), and could introduce bias [20]. For example, both rain and sprinklers can cause a football field to be wet. If one knows the grass is wet, then knowing the sprinklers were off improves your assessment of the probability that it rained; rain and sprinklers become associated when the common effect of "field wetness" is known. Consider a second example from the health sciences: both a thrombus and a haemorrhage can cause a stroke. If we condition on the patient having symptoms of a stroke and learn that there was no haemorrhage, the probability that a thrombotic event occurred is increased. By connecting the two parents of a common child in the figure after Steps 1–3 are completed, we are explicitly stating that we understand that these variables are associated because we have either conditioned on the value of the child or one of the child's descendants (otherwise the variable would have been removed in Step 2). As we shall later see, it is this conditional association that can cause the introduction of bias when traditional rules of confounding adjustment are applied without reference to a DAG. In DAG terminology, the child is called a "collider" because two directed arrows collide at the covariate (node).

### Step 5 (figure 4a): Strip all arrowheads from lines

**Figure 4:**
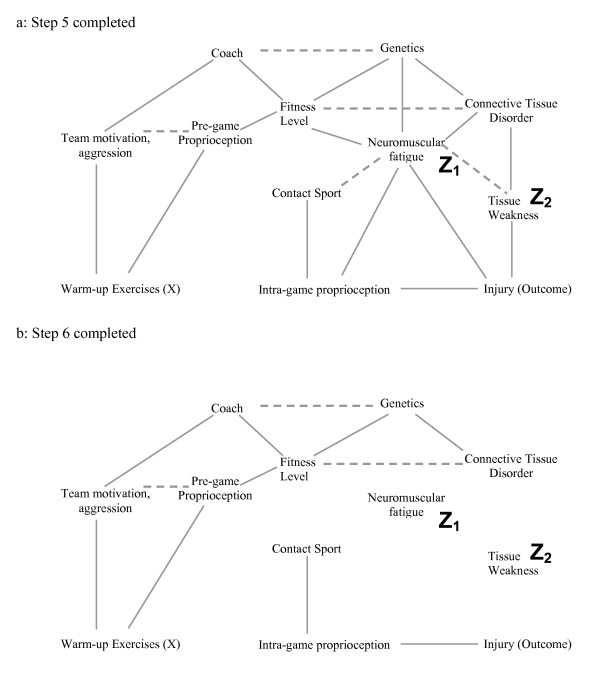
**a-b. In Step 5 (4a), we strip all the arrowheads off all the lines.** In Step 6 (4b), all lines touching the covariates neuromuscular fatigue (Z_1_) and tissue weakness (Z_2_) are deleted. Because the exposure of interest (warm up exercises) is dissociated from the Outcome (injury) after Step 6, the statistical model that includes the covariates neuromuscular fatigue and tissue weakness minimizes the potential bias for the estimate of effect of warm up exercises on the risk of injury.

In Step 5, we strip all the arrowheads from the lines. This is because the arrowheads (causal direction) were only necessary to note the conditional associations created between two parents of a collider. Once this is done, we can simplify the diagram as we have now completed all the steps related to causation.

### Step 6 (figure 4b): Delete all lines between the covariates in the model and any other variables

#### All lines into and out of Neuromuscular fatigue (Z_1_) and tissue weakness (Z_2_) are deleted

Step 6 is simply the graphical equivalent of standard regression techniques. When a covariate is included, the estimate of the effect represents the relationship between the exposure and the outcome independent of any causal pathway going through that covariate; including the covariate "blocks" all associations occurring through this pathway. Therefore, we can delete all lines between the covariates included in the model and any other covariates.

### Interpretation: If X is dissociated from the outcome after Step 6, then the statistical model chosen (i.e. one that includes only the chosen covariates) will minimize the bias of the estimate of X on the chosen outcome

#### If this causal model is correct, then a statistical model that includes a measure of tissue weakness and neuromuscular fatigue minimizes the bias in the estimate of the effect of warming up on the risk of injury

We have now deleted all the direct causal pathways between the exposure of interest and the outcome, and between the covariates and the outcome, and explicitly noted the conditional associations created by including specific covariates with two different causes as explained in step 4. If there is no uninterrupted series of lines through nodes from X to the outcome after completing the six steps (figure 4b), then within this specific causal DAG, there is no non-causal structural association between X and the outcome. In other words, any measured association between the exposure and outcome that exists conditional on the covariates in the model minimizes the bias in the estimate of the causal relationship.

## Discussion

### When including covariates creates a conditional association and introduces bias

**Figure 5:**
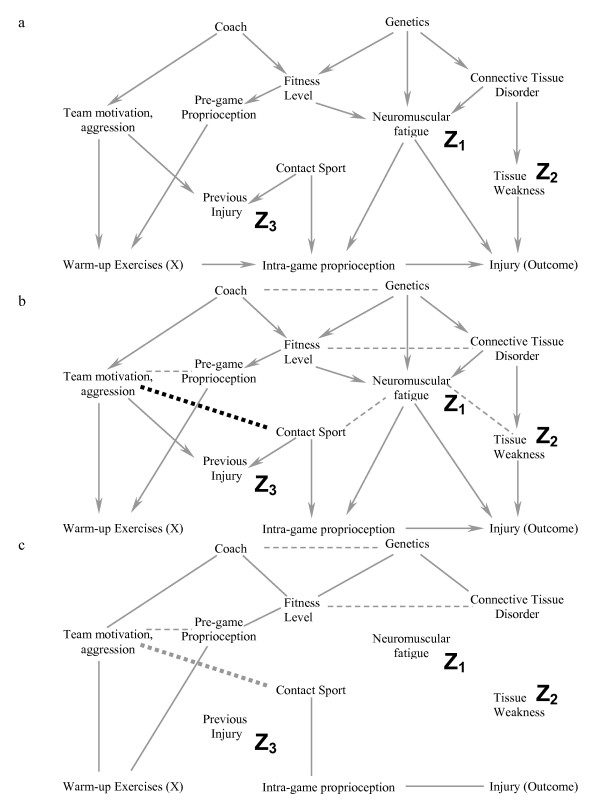
**a-c. This example illustrates the effect of adding the covariate "previous injury" (Z_3_) to the statistical model used for the causal diagram in Figure 2a.** Note that previous injury is associated with both warming up (through team motivation/aggression) and the outcome injury (through Contact Sport). After completing steps 1–4, one is left with figure 5b. Because previous injury (Z_3_) is included in the model, it has not been deleted from the causal diagram in Step 2, and one must join its ancestors (dotted line). Figure 5c represents the causal diagram after completing Steps 5–6. Because warm up is not dissociated from the outcome risk of injury in figure 5c, the statistical model that includes the covariates Z_1_, Z_2_, and Z_3 _will yield a biased estimate of warm up on the risk of injury.

In the last step of this process, we show that including a different subset of covariates in the statistical model can introduce a conditional association or bias (called "collider-stratification bias" or "selection bias" by different authors) (figure 5). In this example, we again include neuromuscular fatigue (Z_1_) and tissue weakness (Z_2_), and add the covariate previous injury (Z_3_) to our statistical model. Note that previous injury is a marker for a direct cause of warming up (X) (team motivation/aggression). It is also a marker for contact sport (an indirect cause of the outcome). Therefore previous injury is associated with both the exposure and the outcome and many researchers would include it in the statistical model. Figure 5a–c show the result of including previous injury in the model graphically. The key to the process in this case lies in step 4. Because previous injury is now present in the model, its two parents are conditionally associated (because including Z_3 _means the value of Z_3 _is known) where they were not associated in the previous example. After step 6, warming up remains connected to the outcome and therefore the estimate of the effect of warming up on the injury would be biased. It is essential to understand that previous injury (Z_3_) may be a very important predictor of the outcome, and techniques such as stepwise regression might strongly suggest that it be included in the model. Further, simply measuring univariate relationships and finding that Z_3 _is related to both the exposure and the outcome would also suggest that it be included in the model. Finally, adding Z_3 _to a model that included Z_1 _and Z_2 _would indeed change the effect estimate, and this is often used as a criterion to suggest that a specific covariate causes confounding bias. It is only through an understanding of the theoretical framework that one realises that including Z_3 _in the model along with Z_1 _and Z_2 _will lead to a conditional association and a biased estimate of effect.

Understanding the conditional associations naturally leads to what is sometimes known as the structural approach to bias [5,15]. Using this approach, epidemiologic biases can be categorized as either lack of conditioning on a common cause (known as confounding bias), or conditioning on a common effect of two parents (or a descendant of the common effect; known as selection bias). The typical selection bias described in observational studies is due to conditioning on a common effect (one conditions on willingness to participate), as are Berkson's bias (conditioning on admission to hospital), loss to follow-up or missing data (conditioning on presence of data; occurs in both observational or randomized trials), some forms of Simpson's Paradox, etc [5,12]. Indeed, we believe it is possible to represent all epidemiologic biases in DAGs; therefore, the restrictions we set out at the beginning of this article concerning an ideal study were used only as a pedagogical tool and are not necessary for this approach.

Selecting a subset of covariates that minimizes the bias in the estimate of the effect requires trial and error and a sound foundation of the theoretical model. At the present time, there is no algorithm and the six-step process should be repeated until a subset of covariates is found such that X is dissociated from the outcome after the 6-step process is completed.

### Additional Advantages

There are two other potential advantages to the DAG approach. First, only a subset of covariates that are associated with both exposure and outcome are necessary to yield an unbiased estimate of effect. Second, because one may require fewer covariates in the model, the statistical efficiency of the analysis is increased (i.e. there are more degrees of freedom if one uses fewer covariates).

### Limitations to the 6-Step approach

The immediate question that always arises is how can one know the true underlying causal structure in order to draw the DAG (i.e. Step 0) – if we knew it, we wouldn't have to study the disease. Although it can be a challenging exercise, the fact remains that understanding the causal structure is an essential step when one wants to know if including a covariate is likely to reduce or increase bias in the effect estimate. In other words, the DAG representing the true causal structure exists even if we do not know what it is, and all causal inferences based on statistical models are implicitly based on a causal structure – the DAG approach simply makes the assumptions explicit.

**Figure 6:**
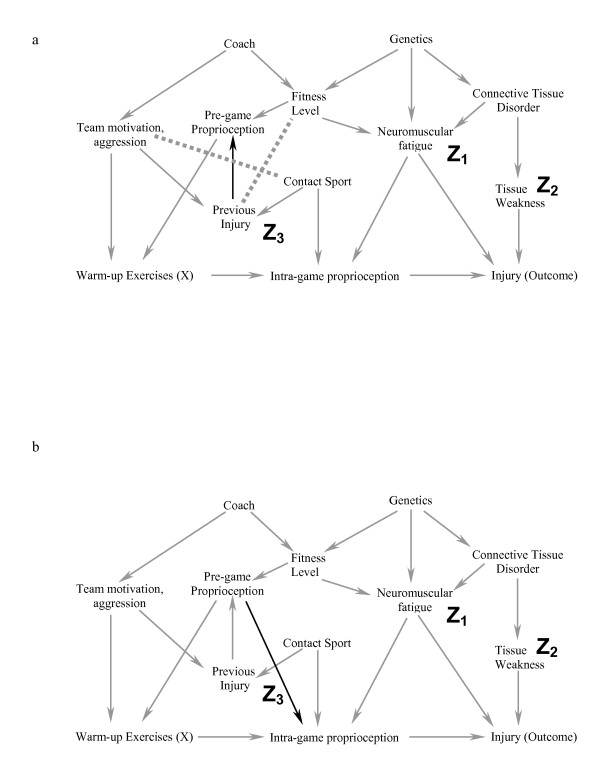
**a-b. Figure 6a is an example of an alternative causal diagram to figure 2a.** The only difference between the two is an additional causal relationship where previous injury causes a decrease in pre-game proprioception (we have also included the additional conditional associations that occur as a result of this change with dotted lines). We are still interested in the causal effects of warm-up on injury risk. Because previous injury is an ancestor of warm up exercises (previous injury causes a decrease in pre-game proprioception which causes an increase in warm up exercises), it is not deleted in Step 2. This leads to two effects. First, contact sport is now a common cause of exposure and outcome. Second, there are additional conditional associations in Step 4 (dotted lines) even if "Previous Injury" is not conditioned on in the statistical model because one is already conditioning on a descendant of previous injury (i.e. the main exposure of interest, warm-up); the effect estimate of warm-up on injury is biased if the statistical model includes only warm-up, neuromuscular fatigue and tissue weakness. Figure 6b shows the same causal diagram as 6a (without the conditional associations), but now a causal link is added from pre-game proprioception to intra-game proprioception.

**Figure 7:**
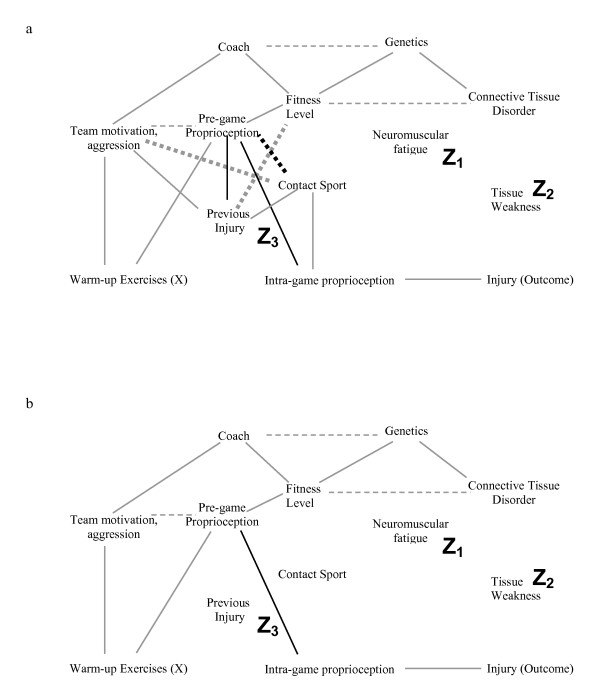
**a-b. Figure 7a represents the causal diagram in Figure 6b after step 5 (dark dotted line represents the additional conditional association due to the new causal link in figure 6b), and Figure 7b shows the result after step 6 if one conditions on Tissue Weakness, Neuromuscular Fatigue, Previous Injury and Contact Sport.** The presence of a path through the variables Warm-up Exercise, Pre-game proprioception (directly, or indirectly through Team Motivation/Aggression) and Intra-game proprioception to Injury means that we would still obtain a biased estimate for the causal effect of warm-up on the risk of injury.

As an example, the causal DAG in Figure 2a may be incorrect and one alternative is illustrated in Figure 6a. In this causal diagram, we have added a causal link from previous injury to pre-game proprioception, and indicated the additional conditional associations that occur due to this change using dotted lines. If Figure 6a represents the true causal diagram, traditional regression/stratification using only neuromuscular fatigue and tissue weakness with or without previous injury will introduce bias for the following reason. Previous injury is now an ancestor of warm-up exercises (previous injury causes pre-game proprioception which causes warm-up) and is therefore not deleted in Step 2 and this leads to two important features. First, contact sport is now a common cause of warm-up (contact sport – previous injury – pre-game proprioception – warm-up) and of injury (contact sport – intra-game proprioception – injury) and therefore including only neuromuscular fatigue and tissue weakness will still provide a biased estimate. Second, the conditional association between Team Motivation/Aggression and Contact Sport exists whether or not we condition on previous injury because we have already conditioned on a descendant of previous injury in this DAG (i.e. warm-up). Therefore, although adding previous injury or pre-game proprioception to the statistical model would block the bias due to the common cause "contact sport", the inclusion of either of these variables does not block the conditional association that now exists between Team motivation/Aggression and Contact Sport; using the six-step algorithm illustrates this clearly for those not used to working with DAGs. In Figure 6b, we present a different causal diagram where we have added a causal link from pre-game proprioception to intra-game proprioception. Figure 7a shows the diagram after step 4 has been completed, and Figure 7b shows the result after completing all the algorithmic steps once we condition on Tissue Weakness, Neuromuscular Fatigue, Previous Injury and Contact Sport. The presence of a path through the variables Warm-up Exercise, Pre-game proprioception (directly or through Team Motivation/Aggression), and Intra-game proprioception to Injury means that we still have a biased estimate. Authors who make causal inferences without explicitly using the DAG approach are assuming a specific DAG (i.e. causal structure) without consideration of other possibilities.

Drawing causal DAGs can be challenging. Causal DAGs represent theory, and theory needs to be developed within the context of all the evidence (basic science, observational and clinical trials) available. Because of this, generating a causal DAG necessarily requires the collaboration of methodological experts, clinicians, physiologists, and others (e.g. psychologists, sociologists) depending on the particular question. The inclusion of latent (unmeasured) variables poses additional problems [21,22]. For many conditions, it is likely that even after reviewing all the evidence, we still won't have enough information to determine if one particular DAG is more appropriate than another DAG. Under these conditions, it is necessary to draw each of the possible DAGs and determine if the same choice of covariates yields an unbiased estimate for each. If not, then one should present each of the interpretations and future research will determine which causal diagram, and which interpretation is correct. Not using the causal approach because of uncertainty on which is the correct DAG simply means that one is allowing chance rather than rational deliberation to make the choice among the different causal diagrams. A further corollary of the structural approach to bias is that an understanding of biological mechanisms and basic science is necessary for appropriate epidemiological studies, and that cross-discipline collaborations should be encouraged.

The DAG approach requires a "node" for each of the covariates. Effect modifiers or covariates that interact with other covariates in a synergistic or antagonistic manner are not currently indicated as such in a DAG. Although there is some theoretical work currently being done in this area (e.g. [23,24]), one can conceptually think of two binary variables that interact as a single variable with multiple levels, and include them as a single node in the DAG. This is somewhat analogous to treating socio-economic status as one variable in a model even though it represents the two distinct constructs of sociological and economical influences. As is known, when two variables both cause a third variable, there is interaction on either the multiplicative scale, additive scale, or both. Therefore, if a DAG were to model synergism or antagonism, one would need different DAGs for different measures of effect (e.g. risk ratio versus risk difference). Finally, issues related to sufficient and component causes have also recently been addressed elsewhere [25].

The DAG approach is not a statistical technique that yields an estimate of effect. However, it will allow users of traditional stratification and regression techniques to reduce the magnitude of the bias in the estimate. Although researchers should generally not adjust for a covariate (or a marker for a covariate) that lies along a causal pathway when assessing the total causal effect, this may not be the case for researchers interested in decomposing total causal effects into direct and indirect effects. In these cases, one may sometimes need to include covariates that lie along the causal path, but this is a process that needs to be carefully thought out or incorrect inferences may occur [26,27]. We also think it is important to highlight the effect of newer statistical techniques to assess total causal effect like marginal structural models [28] that are often necessary in special situations, such as when the covariate is affected by exposure or when a covariate is both a "collider" and a "confounder" at the same time [29,30].

## Conclusion

The traditional approach to confounding bias by determining only associations and avoiding discussions related to causation is problematic and has led to inappropriate data analysis and interpretation [10,13]. The DAG approach can be used to help choose which covariates should be included in traditional statistical approaches in order to minimize the magnitude of the bias in the estimate produced. Investigators should become aware of the other statistical causal approaches available so that the appropriate technique is used to answer the appropriate question.

## Competing interest

The authors declare that they have no competing interests.

## Appendix

A short summary of the Six-Step Process Towards Unbiased Estimates

Step 1. The covariates chosen to reduce bias should not be descendants of X

Step 2. Delete all variables that satisfy all the following criteria: 1) non-ancestors of X, 2) non-ancestors of the outcome and 3) non-ancestors of the covariates that one is including in the model to reduce bias.

Step 3. Delete all lines emanating from X.

Step 4. Connect any two parents sharing a common child.

Step 5. Strip all arrowheads from lines.

Step 6. Delete all lines between the covariates in the model and any other covariates

Interpretation: If X is dissociated from the outcome after Step 6, then the statistical model chosen (i.e. one that includes only the chosen covariates) minimizes the bias of the estimate of X on the chosen outcome.

### Glossary of Terms

#### Genealogy: The DAG approach often uses terms familiar in genealogy

1. Parent: A parent is a direct cause of a particular variable.

2. Ancestor: An ancestor is a direct cause (i.e. parent) or indirect cause (e.g. grandparent) of a particular variable.

3. Child: A child is the direct effect of a particular variable, i.e. the child is a direct effect of the parent.

4. Descendant: A descendant is a direct effect (i.e. child) or indirect effect (e.g. grandchild) of a particular variable.

#### Causes, Effects and Associations

1. Common Cause: A common cause is covariate that is an ancestor of two other covariates.

2. Common Effect (also known as collider): A common effect is a covariate that is a descendant of two other covariates. The term collider is used because the two arrows from the parents "collide" at the node of the descendant.

3. Conditioning: Conditioning on a variable means that one has used either sample restriction or stratification/regression (stratification/regression being two forms of the same mathematical approach) to examine the association of exposure and outcome within levels of the conditioned variable. Other terms often used such as "adjusting for" or "controlling for" suggest an interpretation of the statistical model that is sometimes misleading and therefore we prefer the word conditioning.

4. Unconditional Association: If knowing the value of one covariate provides information on the value of the other covariate without conditioning on any other variable, the two variables are said to be unconditionally associated. This is also known as marginal statistical dependence and its absence as marginal statistical independence.

5. Conditional Association: If knowing the value of one covariate provides information on the value of the other covariate after conditioning on one or more covariates (i.e. within any level of the conditioned covariate(s)), the two variables are said to be conditionally associated. This is also known as conditional statistical dependence and its absence as conditional statistical independence.

#### Structural Approach to Bias: Structural sources of bias include [5,15]

1. Confounding bias: occurs when there is a common cause of the exposure and outcome that is not "blocked" by conditioning on other specific covariates.

2. Selection bias: occurs when one conditions on a common effect (e.g. Berkson's Bias, loss to follow-up, missing data, healthy worker bias, etc) such that there is now a conditional association between the exposure and the outcome.

## Authors' contributions

Both IS and RWP contributed to the development of ideas and the writing of the manuscript. All authors have read and approved the final manuscript.

## Pre-publication history

The pre-publication history for this paper can be accessed here:


